# Maternal diet quality with child allergic and respiratory multimorbidity in the Elfe birth cohort

**DOI:** 10.1038/s41598-024-63456-3

**Published:** 2024-06-06

**Authors:** Rosalie Delvert, Marie-Aline Charles, Bénédicte Leynaert, Manik Kadawathagedara, Karine Adel-Patient, Amandine Divaret-Chauveau, Marie-Noëlle Dufourg, Chantal Raherison, Raphaëlle Varraso, Blandine de Lauzon-Guillain, Annabelle Bédard

**Affiliations:** 1 Université Paris-Saclay, UVSQ, Univ. Paris-Sud, Inserm, Équipe d’Épidémiologie respiratoire intégrative, CESP, Villejuif, France; 2 Université Paris Cité and Université Sorbonne Paris Nord, Inserm, INRAE, Centre for Research in Epidemiology and StatisticS (CRESS), Paris, France; 3 Unité Mixte Inserm-Ined-EFS Elfe, Ined, Aubervilliers, France; 4 Université Paris-Saclay, CEA, INRAE, DMTS, SPI/Laboratoire d’Immuno-Allergie Alimentaire, 91191 Gif-Sur-Yvette, France; 5 Unité d’allergologie pédiatrique, Hôpital d’Enfants, CHRU de Nancy, UR3450 DevAH, Université de Lorraine, Vandoeuvre-Lès-Nancy, France; 6 Bordeaux University, Inserm, Bordeaux Population Health Research Center, Team EPICENE, UMR 1219, Bordeaux, France; 7 Service de Pneumologie, CHU de Guadeloupe, Pointe-à-Pitre, France

**Keywords:** Maternal diet, Allergy, Asthma, Prenatal, Clustering, Epidemiology, Epidemiology, Asthma, Risk factors, Paediatric research

## Abstract

Evidence linking maternal diet during pregnancy to allergic or respiratory diseases in children remains sparse, and outcomes were mainly studied separately. We aim to investigate these associations by considering clusters of allergic and respiratory multimorbidity among 9679 mother–child pairs from the Elfe birth cohort. Maternal diet quality was evaluated using a food-based score (Diet Quality score), a nutrient-based score (PANDiet score) and food group intakes. Adjusted multinomial logistic regressions on allergic and respiratory multimorbidity clusters up to 5.5 years were performed. Child allergic and respiratory diseases were described through five clusters: “asymptomatic” (43%, reference), “early wheeze without asthma” (34%), “asthma only” (7%), “allergies without asthma” (7%), “multi-allergic” (9%). A higher PANDiet score and an increased legume consumption were associated with a reduced risk of belonging to the “early wheeze without asthma” cluster. A U-shaped relationship was observed between maternal fish consumption and the “allergies without asthma” cluster. To conclude, adequate nutrient intake during pregnancy was weakly associated with a lower risk of “early wheeze without asthma” in children. No association was found with food groups, considered jointly or separately, except for legumes and fish, suggesting that maternal adherence to nutritional guidelines might be beneficial for allergic and respiratory diseases prevention.

## Introduction

The first 1000 days from conception represent a critical window of risks and opportunities for the future health of the child^1^. Maternal diet during pregnancy has been associated with developmental and long-term health consequences in offspring^2^. This modifiable factor may be an interesting lever to prevent childhood allergic and respiratory diseases^3^. However, studies on maternal diet and allergic and respiratory diseases reported heterogenous results^4^ possibly due to differences in terms of child age, dietary exposure (nutrients, food groups or overall diet) and in the allergic and respiratory diseases considered. Studying the overall diet of pregnant women with scores may help synthesise complex information as nutrients and foods are not consumed in isolation, and this could be complementary to studying food groups separately in order to help establish nutritional guidelines. A higher Healthy Eating Index (HEI-2015) has been associated with a lower risk of asthma in children^5^, and a low Dietary Approaches to Stop Hypertension (DASH) score with a higher risk of preschool wheezing^6^, but five studies did not report an association between maternal diet quality scores and allergic and respiratory diseases in children^7–11^.

These studies considered allergic and respiratory diseases separately despite their frequent coexistence in individuals^12^. Allergic and respiratory comorbidities are complex and vary among children, reflecting different phenotypes and specific mechanisms. Exploring patterns of coexistence of allergic and respiratory symptoms could offer new insights into these complex diseases. In a recent study involving 1316 mother–child pairs, we reported that infrequent maternal legume consumption was associated with a higher risk of allergic and respiratory multimorbidity in children^7^. However, investigations were limited by the size of the cohort, and these associations need to be further explored in a larger sample with more variability in dietary habits.

We aimed to investigate the associations of the quality of maternal diet during pregnancy with allergic and respiratory multimorbidity in children.

## Methods

### Study design

Elfe (*Etude Longitudinale Française depuis l'Enfance*) is a prospective nationwide birth cohort including 18,329 children born in 2011 from 320 maternity units in mainland France^13^. Inclusion started in April 2011 and was divided in four periods to consider seasonal variability. Singletons or twins born after 33 weeks of gestation whose mothers were aged 18 years or older with no plan to move outside metropolitan France in the following 3 years were eligible^13^. Various types of data were collected from questionnaires completed at inclusion, at several follow-up points and from medical records including socio-demographic, lifestyle, nutritional, and health data.

### Ethics

Enrolled mothers gave written consent for themselves and participation of their child and fathers signed the consent form for the participation of their child or were informed of their right to object.

Ethical approvals were obtained for the Elfe study from the Advisory Committee for Treatment of Health Research Information (Comité Consultatif sur le Traitement de l'Information en matière de Recherche dans le domaine de la Santé), the National Data Protection Authority (Commission Nationale Informatique et Libertés), and the National Committee for Statistical Information. We confirm that all experiments were performed in accordance with relevant guidelines and regulations.

### Data collection

#### Maternal diet quality

Dietary intakes of the mothers during the last three months of pregnancy were collected during maternity stay with a validated semi-quantitative food frequency questionnaire including 125 items designed for French pregnant women^14^. Daily frequency was calculated and multiplied by portion size (in grams) to obtain daily food intakes. Nutritional intakes were obtained by crossing daily food intakes with the SU.VI.MAX food composition database^15^. Questionnaires with more than 10 missing items or with implausible daily energy intake (> 5072 kcal/day (97th percentile) or < 933 kcal/day (3th percentile)) were considered invalid, and mothers were excluded from the study.

The quality of maternal diet during pregnancy was assessed with the Diet Quality score, the Probability of Adequate Nutrient intake Diet quality index (PANDiet score), and 11 food groups. The Diet Quality score^14^, based on French guidelines for pregnant women by food groups^16^, ranges from 0 to 18 points ; a higher score corresponding to a higher diet quality. The PANDiet score, previously adapted for pregnant women, ranges from 0 to 100 points ; a higher score reflecting a better nutritional adequacy, i.e. better adherence to French dietary reference values for pregnant women^17^. Scores were considered continuously, and food groups were considered as continuous, dichotomous or 3-class categorical variables according to French nutritional guidelines^16^ (Supplementary Table S1).

#### Allergic and respiratory variables

Information on allergic and respiratory health of included children were collected by repeated phone interviews to parents. For this study, we used the following synthetic variables in children followed up to age 5.5: ever food allergy, ever itchy rash, ever night cough, ever wheezing, ever medication for asthma, ever medical consultation for asthma, ever allergic conjunctivitis, and allergic rhinitis at 5.5 years. “Ever” outcomes were defined by at least one report of the mentioned health event during follow-up (Supplementary Table S2).

#### Study sample

**Figure 1:**
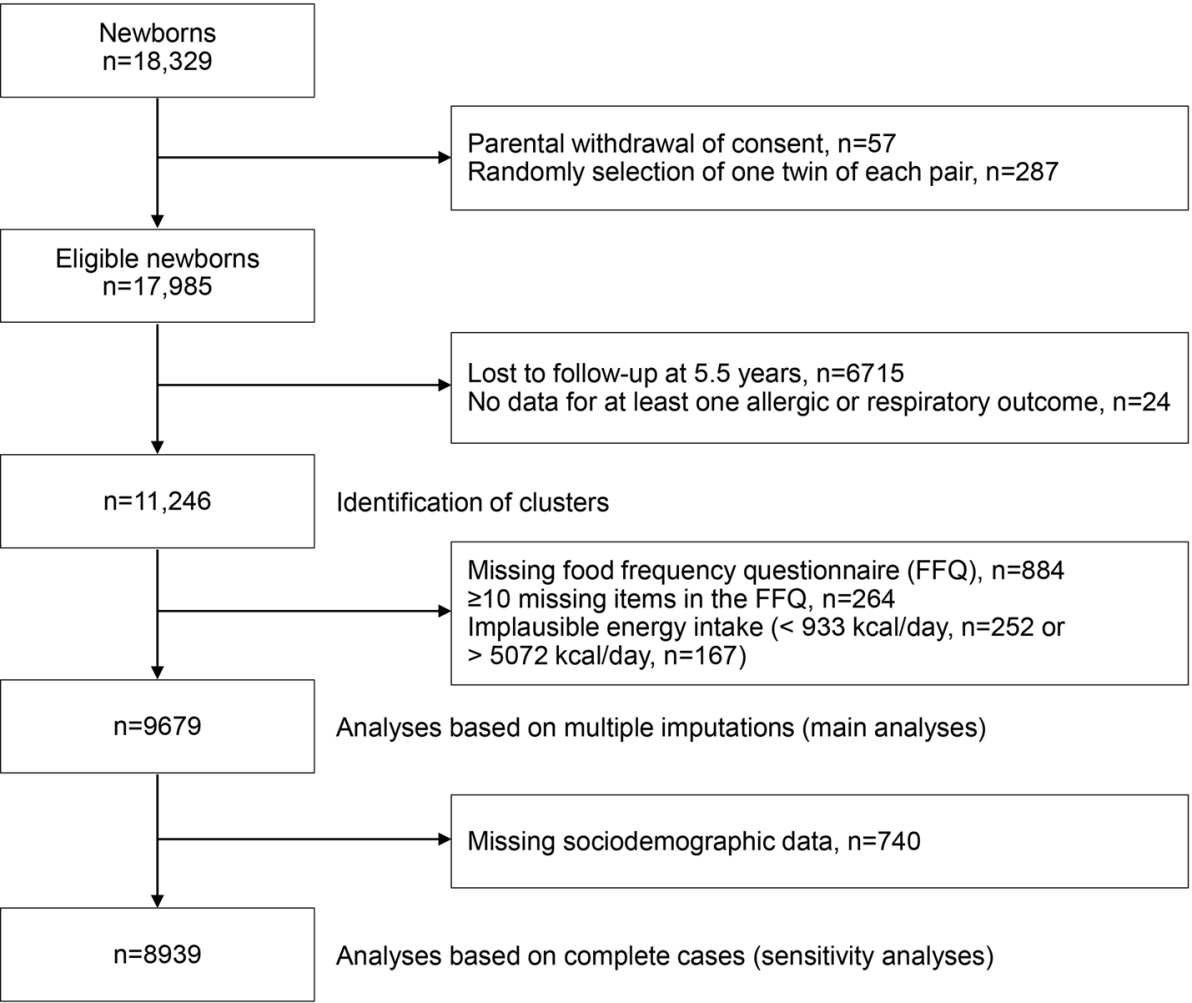
Flowchart of the study population.

Among the 18,329 mother–child pairs of the Elfe birth cohort, 57 were excluded after parents withdrew consent and one twin of two was randomly excluded to avoid family dependence (n = 287). Data for cluster construction were available for 11,246 children. When dietary information was missing or incorrectly completed, mothers were excluded (n = 1567). Finally, 9679 mother–child pairs were included in the main analyses (Fig. 1).

### Statistical analysis

Included mothers were compared to excluded ones on baseline characteristics with Student t-test or Pearson chi-square test.

Allergic and respiratory multimorbidity clusters up to 5.5 years were determined using Latent Class Analysis (LCA) on the 8 synthetic variables mentioned before (ever food allergy, ever itchy rash, ever night cough, ever wheezing, ever medication for asthma, ever medical consultation for asthma, ever allergic conjunctivitis, allergic rhinitis). Solutions of 2 to 6 clusters were tested with 100 replications and 100,000 iterations. The optimal number of clusters was selected through Bayesian Information Criterion (BIC) minimization and interpretability.

Separate multinomial logistic regressions were run for each dietary variable in unadjusted and adjusted analyses. The potential confounders were selected through a directed acyclic graph (Supplementary Figure S1). Models were then adjusted for maternal characteristics (age, education level, household income, living area, region, migration status, smoking status during pregnancy, pre-pregnancy body mass index, physical activity during pregnancy, parity and total energy intake), child characteristics (family history of atopy (i.e. asthma, allergic rhinitis, eczema in parents or siblings), sex), and variables related to study design (period of enrolment, corresponding to season of birth, size of maternity unit). No interaction was found between dietary variables and family history of atopy, child’s sex, or maternal smoking status during pregnancy. For multiple categorical variables, *p*-trend or quadratic trend *p*-values were calculated. Multiple imputation on missing covariates was performed (Supplementary Table S3). Analyses in the imputed dataset are presented as main analyses and were replicated in the complete-case sample to assess consistency.

To deal with potential selection and attrition bias, sensitivity analyses were performed based on weighted data. Calculation of weights included the inclusion procedure, biases related to non-consent or attrition^18^ and calibration on margins from the state register's statistical data and the 2010 French National Perinatal study^19^ on region, age, migration status, education level, marital status and primiparity. Results on food groups were corrected for multiple testing using the False Discovery Rate procedure^20^, a q-value < 0.1 was considered significant.

All analyses were performed with SAS version 9.4 (SAS Institute Inc, Cary, NC, USA), except LCA performed with R software version 4.1.2 (R Foundation for Statistical Computing, Vienna, Austria).

## Results

### Sample characteristics

Included mother and child pairs differed from excluded pairs particularly in terms of age, socioeconomic level, migration status and family history of atopy (Table 1). In our study population the Diet Quality Score ranges between 8.8 and 17.0 points, and the PANDiet score between 23.7 and 88.1 points; maternal diet during pregnancy is described in Table 2 and Supplementary Table S4.

**Table 1:** Characteristics of included and excluded mother–child pairs.

	Excluded sample n = 8650		Included sample n = 9679		*p*-value^¶^
	Missing % (n)	% (n) or mean ± SD	Missing % (n)	% (n) or mean ± SD	
Maternal age at delivery (years)	4.6% (397)	29.9 ± 5.4	0.0% (1)	31.6 ± 4.6	< 0.001
Maternal education level	29.7% (2572)		2.8% (275)		< 0.001
Upper to lower secondary		6.9% (417)		2.1% (195)	
Upper secondary		46.9% (2849)		27.3% (2565)	
Intermediate		19.2% (1169)		25.1% (2365)	
3-year university degree		13.0% (792)		21.2% (1993)	
At least 5-year university degree		14.0% (851)		24.3% (2286)	
Household income^†^ (euros/month)	30.8% (2661)	1448 ± 1084	3.9% (375)	1755 ± 941	< 0.001
Living in an urban area	4.0% (350)	84.4% (7005)	0.0% (0)	75.3% (7291)	< 0.001
Maternal migration status	23.6% (2044)		1.5% (141)		< 0.001
Immigrant		14.6% (967)		7.2% (685)	
Second generation immigrant		12.2% (807)		9.3% (883)	
Born in France to French parents		73.1% (4832)		83.6% (7970)	
Smoking during pregnancy	6.4% (557)		0.8% (79)		< 0.001
Never smoker		55.9% (4521)		58.2% (5583)	
Smoker before pregnancy		19.2% (1555)		25.7% (2464)	
Smoker in early pregnancy		4.5% (361)		3.6% (341)	
Smoker throughout pregnancy		20.5% (1656)		12.6% (1212)	
Pre-pregnancy BMI (kg/m^2^)	7.1% (612)	23.7 ± 5.0	1.0% (97)	23.3 ± 4.6	< 0.001
Number of older children	22.4% (1941)		1.1% (110)		< 0.001
No siblings		46.4% (3115)		43.1% (4129)	
One sibling		32.8% (2200)		39.1% (3740)	
At least 2 siblings		20.8% (1394)		17.8% (1700)	
Season of birth	4.0% (344)		0.0% (0)		< 0.001
Spring–summer		38.6% (3207)		42.1% (4070)	
Fall-winter		61.4% (5099)		58.0% (5609)	
Boys	4.5% (386)	52.0% (4680)	0.0% (0)	50.9% (4553)	0.07
Family history of atopy	29.0% (2507)	44.8% (3014)	1.6% (150)	53.1% (4744)	< 0.001
Gestational age (weeks)	6.2% (535)	39.1 ± 1.5	1.4% (134)	39.3 ± 1.4	< 0.001
Child’s birth weight (g)	6.8% (591)	3289 ± 505	1.9% (186)	3350 ± 474	< 0.001

BMI: body mass index ; SD: standard deviation.

^†^by consumption unit ; ^¶^ *p*-value for comparison between included and excluded samples using Student *t* tests for continuous variables and chi-square tests for categorical variables.

**Table 2:** Maternal dietary characteristics of the study population (n = 9679).

	Study population
	n = 9679% (n) or mean ± SD
Diet Quality score (per unit)	13.0 ± 1.2
PANDiet score (per unit)	55.8 ± 9.1
Fruits (times/day)	1.4 ± 1.5
Vegetables (times/day)	1.6 ± 1.2
Legumes	
< 1 time/month	82.0% (7938)
1–4 times/month	13.9% (1347)
> 1 times/week	4.1% (394)
Starch and grains (times/day)	2.9 ± 1.2
Nuts	
No consumption	68.0% (6582)
Consumption	32.0% (3097)
Milk and dairy products	
< 3 times/day	49.6% (4797)
3–4 times/day	23.6% (2287)
> 4 times/day	26.8% (2595)
Fish and shellfish	
< 1 time/week	46.7% (4516)
1–2 times/week	26.8% (2592)
> 2 times/week	26.6% (2571)
Red meat	
< 500 g/week	73.4% (7101)
≥ 500 g/week	26.6% (2578)
Processed meat	
< 150 g/week	81.9% (7927)
≥ 150 g/week	18.1% (1752)
Poultry (g/week)	150.7 ± 157.7
Sweetened beverages (ml/day)	459.6 ± 585.8
Total energy intake (kcal/day)	2166 ± 744

SD: standard deviation.

### Identification of allergic and respiratory clusters

A five-class solution was identified, clusters were labelled according to their allergic and respiratory characteristics (Table 3; Supplementary Table S5):

- “asymptomatic” (43% of children): low prevalence of allergic and respiratory disorders.
- “early wheeze without asthma” (34% of children): high prevalence of respiratory symptoms without medical consultation for asthma. Detailed data showed a high prevalence of wheezing, medication for asthma and night cough until 2 years old which lowered afterwards (50.7% children with reported wheezing at 1 year and 18.5% at 3.5 years).
- “asthma only” (7% of children): high prevalence of respiratory symptoms, but low prevalence of food allergy, rash and allergic conjunctivitis.
- “allergies without asthma” (7% of children): high prevalence of food allergies, rash, allergic conjunctivitis and rhinitis, but low prevalence of asthma and wheezing after 1 year.
- “multi-allergic” (9% of children): high prevalence of multiple allergic and respiratory symptoms.

**Table 3:** Prevalence of allergic and respiratory diseases in children up to 5.5 years in the allergic and respiratory multimorbidity clusters (n = 11,246).

	Total	Allergic and respiratory multimorbidity clusters				
		Asymptomatic	Early wheeze without asthma	Asthma only	Allergies without asthma	Multi-allergic
	n = 11,246	n = 4841	n = 3785	n = 829	n = 804	n = 987
Ever food allergy (0–5.5 years)	7.4% (837)	4.2% (205)	5.2% (195)	0.4% (3)	18.7% (150)	28.8% (284)
Ever itchy rash (0–5.5 years)	53.6% (6028)	45.5% (2201)	53.2% (2012)	35.6% (295)	75.4% (606)	92.6% (914)
Ever wheezing (0–5.5 years)	45.3% (5093)	2.6% (128)	79.5% (3010)	91.9% (762)	31.5% (253)	95.2% (940)
Ever night cough (0–5.5 years)	82.3% (9260)	65.6% (3178)	96.6% (3655)	92.3% (765)	84.6% (680)	99.5% (982)
Ever medication for asthma (0–3.5 years)	40.7% (4581)	3.3% (159)	69.8% (2642)	95.2% (789)	15.4% (124)	87.8% (867)
Ever consultation for asthma (2–5.5 years)	15.7% (1771)	0.7% (33)	0.1% (2)	100.0% (829)	15.9% (128)	78.9% (779)
Ever allergic conjunctivitis (3.5–5.5 years)	36.7% (4130)	28.7% (1391)	34.7% (1314)	26.8% (222)	62.7% (504)	70.8% (699)
Allergic rhinitis (5.5 years)	12.1% (1366)	2.7% (130)	0.7% (25)	11.8% (98)	76.5% (615)	50.5% (498)

Values are in % (n) and are the prevalence of the variable included in the cluster construction.

### Maternal diet and allergic and respiratory multimorbidity clusters

The “asymptomatic” cluster was the reference group for all analyses.

**Overall diet quality**: No association was found between an increment of one unit of the Diet Quality Score and the allergic and respiratory multimorbidity clusters in both unadjusted and adjusted multinomial logistic regressions (Table 4). A borderline association was observed between a 10-point increase of the PANDiet score and a lower risk of belonging to the “early wheeze without asthma” cluster (adjusted odds-ratio; aOR [95%CI] = 0.95 [0.90;1.00]). Such trend was also observed in the analyses based on complete cases (aOR [95%CI] = 0.95 [0.90;1.01]; Supplementary Table S6) and in the weighted analyses (aOR [95%CI] = 0.94 [0.87;1.00]; Supplementary Table S7).

**Table 4:** Associations of maternal diet quality scores during pregnancy with allergic and respiratory multimorbidity clusters in children (n = 9679).

	Allergic and respiratory multimorbidity clusters			
	Early wheeze without asthma	Asthma only	Allergies without asthma	Multi-allergic
Unadjusted models				
Diet Quality score (per point)	0.97 [0.94;1.01]	0.97 [0.91;1.03]	0.97 [0.91;1.04]	1.01 [0.95;1.07]
PANDiet score (per 10 points)	**0.94 [0.90;0.99]**	0.96 [0.88;1.05]	0.98 [0.90;1.08]	1.02 [0.94;1.11]
Adjusted models^†^				
Diet Quality score (per point)	0.97 [0.94;1.01]	0.98 [0.92;1.06]	1.01 [0.94;1.08]	1.03 [0.96;1.09]
PANDiet score (per 10 points)	0.95 [0.90;1.00]	0.99 [0.90;1.09]	0.99 [0.90;1.10]	1.05 [0.96;1.14]

Cluster of reference: “asymptomatic”.

Results with *p*-value < 0.05 are in bold text.

OR [95%CI] from multinomial logistic regressions. OR, odds ratio; CI, confidence interval. Each dietary score was considered in a separate model. Cluster of reference: “asymptomatic”.

^†^Models are adjusted for maternal characteristics (age at delivery, education level, household income, migration status, smoking status during pregnancy, pre-pregnancy body mass index (BMI), physical activity during pregnancy, number of older children, living area (urban/rural), region and total energy intake), child characteristics (sex and family history of atopy), and study design characteristic (period of enrolment, size of maternity unit).

**Food groups intake**: Frequent maternal consumption of legumes was associated with a lower risk of belonging to the “early wheeze without asthma” cluster (aORs [95%CI] = 0.86 [0.75;0.98] and 0.82 [0.64;1.04] for legume consumption 1–4 times/month and > 1 times/week vs. < 1 time/month respectively ; *p* for trend = 0.005) (Table 5). A U-shaped relationship between maternal fish consumption and a higher risk of belonging to the “allergies without asthma” cluster was observed: both low and high consumption were associated with a higher risk than intermediate consumption (< 1 time/week vs. 1–2 times/week: aOR [95%CI] = 1.17 [0.95;1.46]; > 2 times/week vs. 1–2 times/week: aOR = 1.37 [1.08;1.74]; *p* value for U-shape tendency = 0.02); low consumption was significant only in the unadjusted results. No association was found with other food groups or clusters in the main analyses. None of the associations remained significant after correction for multiple testing (Supplementary Table S8).

**Table 5:** Associations of maternal food groups consumption with allergic and respiratory multimorbidity clusters in children (n = 9679).

Model^†^	Food groups	Allergic and respiratory multimorbidity clusters			
		Early wheeze without asthma	Asthma only	Allergies without asthma	Multi-allergic
M0	Fruits (times/day)	**0.96 [0.93;0.99]**	0.98 [0.93;1.03]	0.99 [0.94;1.04]	0.96 [0.92;1.01]
M1	Fruits (times/day)	0.97 [0.94;1.00]	1.00 [0.95;1.06]	1.00 [0.94;1.06]	0.96 [0.90;1.01]
M0	Vegetables (times/day)	0.99 [0.95;1.03]	0.98 [0.92;1.05]	1.01 [0.94;1.07]	0.99 [0.93;1.05]
M1	Vegetables (times/day)	1.01 [0.97;1.05]	1.01 [0.93;1.08]	1.04 [0.96;1.11]	0.98 [0.92;1.05]
M0	Legumes				
	< 1 time/month	1 [Ref]	1 [Ref]	1 [Ref]	1 [Ref]
	1–4 times/month	**0.84 [0.74;0.96]**	0.88 [0.69;1.11]	0.91 [0.72;1.15]	0.92 [0.74;1.13]
	> 1 times/week	**0.75 [0.59;0.96]**	1.02 [0.70;1.50]	1.01 [0.68;1.50]	0.80 [0.54;1.18]
M1	Legumes^¶^				
	< 1 time/month	1 [Ref]	1 [Ref]	1 [Ref]	1 [Ref]
	1–4 times/month	**0.86 [0.75;0.98]**	0.89 [0.70;1.14]	0.92 [0.72;1.18]	0.91 [0.73;1.13]
	> 1 times/week	0.82 [0.64;1.04]	1.11 [0.75;1.65]	0.99 [0.66;1.49]	0.80 [0.54;1.20]
M0	Starch and grains (times/day)	0.99 [0.95;1.03]	0.98 [0.91;1.05]	1.00 [0.93;1.07]	0.98 [0.92;1.05]
M1	Starch and grains (times/day)	0.99 [0.95;1.04]	0.97 [0.89;1.05]	1.01 [0.93;1.09]	0.96 [0.89;1.03]
M0	Nuts (consumption vs never)	0.97 [0.88;1.07]	1.00 [0.85;1.19]	0.88 [0.74;1.05]	**0.85 [0.73;0.99]**
M1	Nuts (consumption vs never)	0.96 [0.86;1.06]	0.99 [0.83;1.18]	0.94 [0.79;1.12]	0.86 [0.73;1.01]
M0	Milk and dairy products				
	< 3 times/day	0.96 [0.86;1.08]	0.85 [0.70;1.03]	1.19 [0.96;1.47]	1.01 [0.84;1.22]
	3–4 times/day	1 [Ref]	1 [Ref]	1 [Ref]	1 [Ref]
	> 4 times/day	0.97 [0.85;1.10]	0.91 [0.73;1.13]	1.00 [0.79;1.27]	1.08 [0.88;1.33]
M1	Milk and dairy products				
	< 3 times/day	0.97 [0.86;1.10]	0.84 [0.68;1.03]	1.16 [0.93;1.44]	0.99 [0.81;1.20]
	3–4 times/day	1 [Ref]	1 [Ref]	1 [Ref]	1 [Ref]
	> 4 times/day	0.98 [0.85;1.12]	0.93 [0.74;1.18]	0.98 [0.76;1.25]	1.06 [0.85;1.32]
M0	Fish and shellfish				
	< 1 time/week	1.03 [0.92;1.15]	1.01 [0.84;1.23]	**1.26 [1.02;1.56]**	0.88 [0.73;1.05]
	1–2 times/week	1 [Ref]	1 [Ref]	1 [Ref]	1 [Ref]
	> 2 times/week	0.97 [0.86;1.10]	0.85 [0.68;1.06]	**1.37 [1.09;1.73]**	0.94 [0.77;1.14]
M1	Fish and shellfish‡				
	< 1 time/week	1.04 [0.93;1.16]	1.03 [0.84;1.25]	1.17 [0.95;1.46]	0.86 [0.71;1.03]
	1–2 times/week	1 [Ref]	1 [Ref]	1 [Ref]	1 [Ref]
	> 2 times/week	1.00 [0.88;1.14]	0.85 [0.68;1.07]	**1.37 [1.08;1.74]**	0.92 [0.75;1.13]
M0	Red meat (≥ 500 vs < 500 g/week)	1.07 [0.97;1.19]	1.06 [0.89;1.27]	1.06 [0.88;1.28]	0.92 [0.78;1.09]
M1	Red meat (≥ 500 vs < 500 g/week)	1.06 [0.95;1.18]	1.04 [0.86;1.26]	1.04 [0.85;1.27]	0.87 [0.73;1.05]
M0	Processed meat (≥ 150 vs < 150 g/week)	1.08 [0.96;1.21]	1.15 [0.94;1.40]	1.17 [0.95;1.44]	1.05 [0.87;1.28]
M1	Processed meat (≥ 150 vs < 150 g/week)	1.05 [0.92;1.19]	1.09 [0.88;1.36]	1.19 [0.96;1.49]	1.01 [0.82;1.24]
M0	Poultry (per 100 g/week)	0.99 [0.97;1.02]	0.95 [0.90;1.01]	1.00 [0.95;1.05]	1.03 [0.99;1.08]
M1	Poultry (per 100 g/week)	1.00 [0.97;1.03]	0.96 [0.90;1.02]	1.00 [0.94;1.05]	1.02 [0.98;1.07]
M0	Sweetened beverages (per 200 ml/day)	0.99 [0.98;1.01]	1.00 [0.97;1.02]	1.02 [1.00;1.05]	1.01 [0.99;1.04]
M1	Sweetened beverages (per 200 ml/day)	0.99 [0.97;1.01]	1.00 [0.96;1.03]	1.01 [0.98;1.04]	1.01 [0.98;1.04]

Cluster of reference: “asymptomatic”.

Results with *p*-value < 0.05 are in bold text.

OR [95%CI] from multinomial logistic regressions. OR, odds ratio; CI, confidence interval. Each dietary exposure was considered in a separate model.

^†^Models M0 are unadjusted, models M1 are adjusted for maternal characteristics (age at delivery, education level, household income, migration status, smoking status during pregnancy, pre-pregnancy body mass index (BMI), physical activity during pregnancy, number of older children, living area (urban/rural), region, total energy intake and PANDiet score), child characteristics (sex and family history of atopy), and study design characteristic (period of recruitment, size of maternity unit).

^¶^*p* for trend = 0.005 ; ‡ *p* for quadratic trend = 0.02.

In the analyses on complete cases, results were not substantially changed (Supplementary Table S6). In the weighted sample analyses, effect estimates for legume and fish consumption remained similar, but no longer significant. Furthermore, starch and grain consumption was related to a lower risk of belonging to the “multi-allergic” cluster and a higher consumption of vegetables was related to a higher risk of belonging to the “allergies without asthma” cluster (Supplementary Table S7).

## Discussion

In this study, allergic and respiratory multimorbidity in children up to age 5.5 was described with five clusters: “asymptomatic”, “early wheeze without asthma”, “asthma only”, “allergies without asthma”, and “multi-allergic”. Overall maternal diet quality during pregnancy was weakly associated with allergic and respiratory multimorbidity clusters in children. A higher consumption of legumes during pregnancy was associated with a lower risk of belonging to the “early wheeze without asthma” cluster in children compared to the “asymptomatic” cluster, and a U-shape association between maternal fish consumption and the “allergies without asthma” cluster was found.

A borderline association between a better nutritional adequacy during pregnancy and a lower risk of early wheeze without asthma in children was observed in our study. In most prior studies, no association was reported for maternal diet quality during pregnancy with allergic and respiratory diseases considered separately^8–11,21,22^ or jointly^7^. However, in an observational study with a 10-year follow-up a higher diet quality was associated with a lower risk of asthma in children^5^. In a pooled analysis from seven European birth cohorts, a very low dietary quality was associated with a higher risk of preschool wheezing^6^, and in two prospective longitudinal studies a healthier diet was associated with a higher risk of eczema at age 1^23^ and 7–9 years^24^, respectively.

In our study, consumption of legumes during pregnancy was associated with a lower risk of early wheeze without asthma in children and was not associated with the other clusters. The association was only significant for moderate consumption (1–4 times/month), and not for the recommended frequency of more than once a week. This may be explained by a lack of statistical power, as only 4% of the population reporting a pluri-weekly consumption of legumes as suggested by the linear trend observed and the unadjusted results. In another cohort, we also highlighted an association between low legume consumption and a higher likelihood of belonging to the “multi-allergic” cluster^7^. In other studies, legumes were associated with a lower risk of eczema^25^ in children, and a borderline association was found with a lower risk of persistent wheeze^26^, but most studies did not report associations with respiratory or allergic symptoms^27–31^. Fibres contained in legumes may explain the lower risks observed. It has been shown that fibres can modify the composition of gut microbiota and protect against airway allergic inflammation^32^. Additionally, maternal transmission likely participates in the establishment of gut microbiota in children^33^. Their potential anti-inflammatory effect may favour the healthy development of the immune system, making children less vulnerable to oxidative stress^6^.

In our study, both a lower and a higher than recommended maternal consumption of fish or shellfish were associated with a higher risk of allergies without asthma in children suggesting a complex interaction between potential beneficial (long-chain polyunsaturated fatty acids) and deleterious (food chemicals) components contained in fish^34^. That association was not found in our previous study on clusters of allergic and respiratory disorders^7^, may be due to a lack of statistical power. In studies considering allergies separately, maternal sea product consumption was associated with a higher risk of eczema^35^, atopy^9^ or food allergy in children^27,36^, but also with a lower risk of eczema^37–40^, allergic rhinitis^37^ or food allergy^41^. A systematic review with a meta-analysis of prospective observational studies reported no association between maternal fish consumption and eczema or wheezing^42^.

Using data from the Elfe birth cohort confers several strengths to our study. The large sample size and the detailed data collected in the Elfe study allowed adjustment on numerous potential confounders, even if residual confounding cannot be excluded. Although comparison between included and excluded participants suggested a selection bias, the design of the Elfe study enables the calculation of weights extending the validity of our results. In weighted analyses, no difference was observed for diet quality scores, but associations were reinforced for starch and grains and vegetables and attenuated for fish and legumes. These discrepancies might be explained by a differential influence of selection bias according to food groups. Maternal dietary exposures were self-reported after delivery which may lead to measurement errors. However, the questionnaire was validated^14^ and focused on the last months of pregnancy to limit recall bias. Our results should not be generalised to the overall pregnancy, even if few changes in dietary intakes have been observed elsewhere between early and late pregnancy^43^. We addressed diet quality during pregnancy with a food-group-based and a nutrient-based score, two complementary approaches, and to identify the possible role of specific food groups, we investigated individual food groups. However, the scores were developed for overall health, not specifically for allergic and respiratory diseases, so the weak associations we found do not mean that maternal diet quality is not important. Breastfeeding and early introduction of food allergens have been associated with the risk of developing atopic diseases^44,45^. However, they have not been considered in our models as potential confounders because they are posterior to maternal diet. Further adjustment for these factors did not substantially change our results (data not shown).

The questionnaire used for allergic and respiratory symptoms was not validated, but is derived from the International Study of Asthma and Allergy in Childhood (ISAAC) questionnaire^46^; misclassification bias cannot be excluded although the prospective design limits recall bias. We accounted for the complex inter-relations between allergic and respiratory diseases in children by constructing unsupervised multimorbidity clusters including most common allergic and respiratory symptoms. Lung function was not available and, our clusters did not provide information on disease course which could be further investigated with longitudinal approaches. In the present study, we identified four clusters similar to those in our previous study performed on a smaller sample size^7^. We also identified an additional cluster of “early wheeze without asthma”, which could correspond to transient wheezing not leading to asthma and which might result from infections since night cough and wheezing are not specific to asthma. This analysis, based on a larger cohort, supports our previous results on diet quality and legumes and provides new elements on fish consumption.

To conclude, in a large birth cohort, allergic and respiratory multimorbidity in children was described with five clusters. While the overall maternal diet quality during pregnancy was weakly associated with allergic and respiratory multimorbidity, a higher legume consumption was associated with reduced risk of early wheeze not leading to asthma in children, and a U-shape relationship between maternal fish consumption and the risk of allergic diseases other than asthma was found. Further studies are needed to confirm if adherence to nutritional guidelines during pregnancy for legumes and fish is beneficial for the prevention of allergic and respiratory diseases in children.

### Supplementary Information

Supplementary Information.

## Data Availability

The data underlying the findings cannot be made freely available for ethical and legal restrictions imposed because this study includes a substantial number of variables that, together, could be used to re-identify the participants based on a few key characteristics and then be used to have access to other personal data. Therefore, the French ethics authority strictly forbids making these data freely available. However, they can be obtained upon request from the Elfe principal investigator. Readers may contact marie-aline.charles@inserm.fr to request the data. The analytic code will be made available upon request pending application and approval.
