# Supplementary material for: Maternal diet quality with child allergic and respiratory multimorbidity in the Elfe birth cohort
Source: Sci Rep. 2024 Jun 6;14:13048. doi: 10.1038/s41598-024-63456-3 (PMC11156635; doi:10.1038/s41598-024-63456-3)
Supplement: Supplementary file 1 — Supplementary Information. [file 41598_2024_63456_MOESM1_ESM.docx]

**ONLINE SUPPLEMENTARY MATERIAL**

**Maternal diet quality with child allergic and respiratory multimorbidity in the Elfe birth cohort**

Rosalie Delvert, Marie-Aline Charles, Bénédicte Leynaert, Manik Kadawathagedara, Karine Adel-Patient, Amandine Divaret-Chauveau, Marie-Noëlle Dufourg, Chantal Raherison, Raphaëlle Varraso, Blandine de Lauzon-Guillain*, Annabelle Bédard*

*The authors contributed equally to this work

**Summary**

[Supplementary Table S1. Composition of food groups included in the data analyses. 3](#_Toc166759930)

[Supplementary Table S2. Details on age at data collection and construction of synthetic allergic and respiratory variables. 5](#_Toc166759931)

[Supplementary Figure S1. Directed Acyclic Graph (DAG) on maternal diet during pregnancy and allergic and respiratory diseases in children, used for the selection of cofounders. 6](#_Toc166759932)

[Supplementary Table S3. Details on multiple imputation (n=9679). 7](#_Toc166759933)

[Supplementary Table S4. Maternal diet during pregnancy according to allergic and respiratory multimorbidity clusters in children (n=9679). 8](#_Toc166759934)

[Supplementary Table S5. Allergic and respiratory characteristics by age of allergic and respiratory multimorbidity clusters (n=11,246). 9](#_Toc166759935)

[Supplementary Table S6. Adjusted associations of maternal diet quality during pregnancy with allergic and respiratory multimorbidity clusters in children from the complete-case sample (n=8939). 10](#_Toc166759936)

[Supplementary Table S7. Weighted associations of maternal diet quality during pregnancy with allergic and respiratory multimorbidity clusters in children from the main sample (n=9677). 11](#_Toc166759937)

[Supplementary Table S8. Adjusted associations of maternal food groups consumption with allergic and respiratory multimorbidity clusters in children (n=9679), after correction for multiple testing. 12](#_Toc166759938)

# Supplementary Table S1. Composition of food groups included in the data analyses.

| **Food groups and frequency assessment** | **Food components** |
| --- | --- |
| Fruits^†^  *per times/day* | Apricots, peaches, plums, cherries; strawberries, raspberries; grapes; melon, watermelon; bananas; kiwis; citrus fruits (oranges, mandarins, grapefruit); apples or pears; exotic fruits (pineapple, mangoes, lychees, guava); dried fruits (dried apricots, dates, figs, prunes) |
| Vegetables^†^  *per times/day* | Green salad, lamb's lettuce, arugula, spinach, watercress; grated carrots; avocado; other raw vegetables (tomatoes, beets, cabbage, cucumber, radish); vegetable soup; green beans; cooked endives, spinach, cress; leeks; cabbage (green, cauliflower, Brussels sprouts, broccoli); cooked carrots; zucchinis, eggplants, peppers, cooked tomatoes (ratatouille); peas; artichokes, fennel, asparagus, celery; corn; pumpkin, sweet potatoes |
| Legumes^¶^  *<1 time/month*  *1-4 times/month*^#^  *>1 times/week* | Lentils, dried beans, chickpeas, broad beans |
| Starch and grains^†^  *per times/day* | Bread (white bread or sandwich bread); wholegrain or other special breads; rusks or crackers or toasts; breakfast cereals (corn flakes, chocolate Cheerios, puffed cereals, muesli); pasta (macaroni, spaghetti, coquillettes); rice; semolina, wheat; boiled or baked potatoes; mashed potatoes; potato gratin; other starches (quinoa, cassava, plantain, yam) |
| Nuts^¶^  *No consumption*  *Consumption* | Walnuts, hazelnuts, almonds; peanuts |
| Milk and dairy products^§^  *<3 times/day*  *3-4 times/day*  *>4 times/day* | Emmental, Gruyère, Comté, Beaufort; Roquefort, blue cheeses; goat cheese; other types of cheeses (Camembert, Brie); cottage cheese or 0% fat yoghurt (plain, with fruits); cottage cheese, petit-suisse cheese or yoghurts with 20%, 30% or 40%; desserts (cream desserts, mousses, flans); ice cream; fresh cream; whole milk; semi-skimmed milk; skimmed milk |
| Fish and shellfish^§^  *<1 time/week*  *1-2 times/week*  *>2 times/week* | Fresh or frozen fish (cod, pollack, whiting, sole, trout); canned fish in oil (tuna, sardines); smoked fish (salmon, trout); salted fish or fish in brine (cod, herring, anchovy); breaded fish; shellfish (mussels, oysters, scallops); shellfish (mussels, oysters, scallops); crustaceans (shrimp, crab) |
| Red meat^¶^  *<500 g/week*  *≥500 g/week* | Beef (excluding ground steak); ground beef steaks; pork (excluding processed meat); veal meat; lamb, mutton meat; liver (heifer, poultry, other); beef tongue, tripe, blood sausage, andouillettes, sweetbreads, kidneys |
| Processed meat^¶^  *<150 g/week*  *≥150 g/week* | Dry sausage (or salami); mortadella, saveloy; pâté, rillettes; ham (white, raw, bacon); fresh or smoked sausages (including merguez) |
| Poultry^†^  *per g/week* | Poultry (chicken, turkey), rabbit |
| Sugar-sweetened beverages^†^  *per ml/day* | Orange juice, grapefruit juice, pineapple juice, apple juice, grape juice; syrup or flavoured water; sugar-sweetened cola, lemonade, or sodas |

Foods separated with a “,” are from the same item, foods separated with a “;” are from different items.

Food groups used as continuous, dichotomous variable, or categorized into 3 classes according to the French nutritional guidelines:

^†^ Food groups were used as continuous variables when the guideline provided qualitative recommendations without specific threshold or optimal value (e.g., “limit”, “increase”)

^¶^ Food groups were used as dichotomous variables when the guideline suggested a specific threshold

^§^ Food groups were categorized into 3 classes when the guideline suggested an optimal value (below optimal value/optimal value/above optimal value)

^#^ For legumes, too few women complied with the recommendation, so an intermediate category has been created.

# Supplementary Table S2. Details on age at data collection and construction of synthetic allergic and respiratory variables.

|  | **2 months** | **1 year** | **2 years** | **3.5 years** | **5.5 years** |
| --- | --- | --- | --- | --- | --- |
| Ever food allergy | x |  | x | x | x |
| Ever itchy rash | x | x | x | x | x |
| Ever night cough | x | x | x | x | x |
| Ever wheezing | x | x | x | x | x |
| Ever medication for asthma |  | x | x | x |  |
| Ever medical consultation for asthma |  |  | x | x | x |
| Ever allergic conjunctivitis |  |  |  | x | x |
| Allergic rhinitis |  |  |  |  | x |

Blank cells indicate that information was not collected at this age.

Ever food allergy: parental report of medical diagnosis of cow’s milk proteins allergy at 2 months, or at least one report of exclusion of certain food due to food allergy on medical advice in the last 12 months between 2 and 5.5 years.

Ever itchy rash: at least one parental report of itchy rash at 2 months or 1 year, or parental report of eczema between 2 and 5.5 years.

Ever night cough: at least one parental report of night cough between 2 months and 5.5 years.

Ever wheezing: at least one parental report of wheezing in the chest between 2 months and 5.5 years.

Ever medication for asthma: at least one parental report of use of bronchodilators or inhaled corticosteroids in the last 12 months between 1 and 3.5 years.

Ever medical consultation for asthma: at least one parental report of a medical consultation for asthma in the last 12 months between 2 and 5.5 years.

Ever allergic conjunctivitis: at least one parental report of eye allergy or allergic conjunctivitis at 3.5 years or 5.5 years.

Allergic rhinitis: ever hay fever or allergic rhinitis reported by parents at 5.5 years.

As allergic rhinitis was collected only at 5.5 years, only children followed until 5.5 years were included in our study population.

**
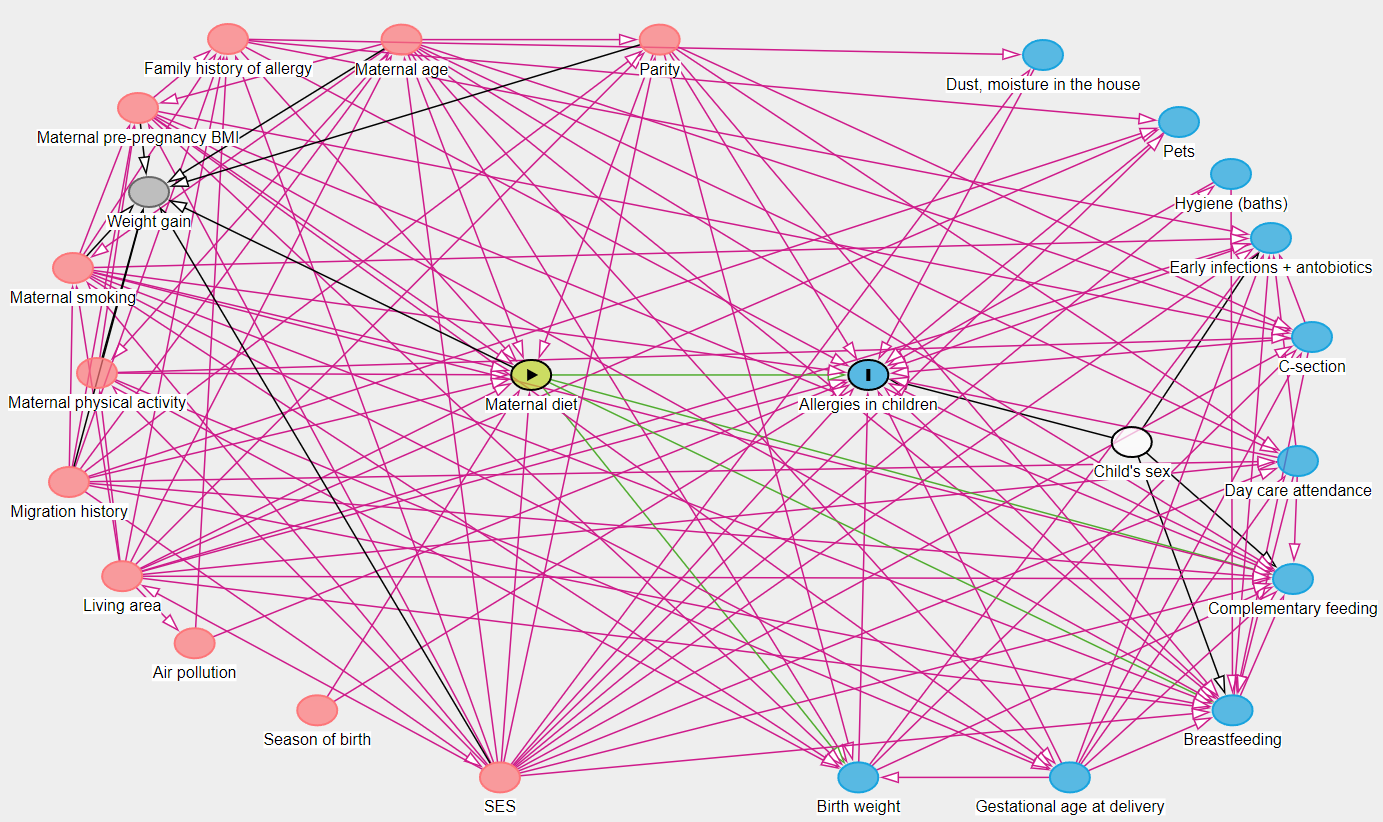
**

# Supplementary Figure S1. Directed Acyclic Graph (DAG) on maternal diet during pregnancy and allergic and respiratory diseases in children, used for the selection of cofounders.

Graph made with DAGitty v3.1. The triangle in a **green circle** represents the **main exposure** (maternal diet during pregnancy) and the I in a **blue circle** is the **outcome** (allergies in children). **Red circles** represent **potential confounders** (i.e. ancestors of exposure and outcome), **intermediate factors** (i.e. ancestor of the outcome but not of the exposure) are represented with **blue circles**. Variables included in the adjustment that are not confounders are represented in a white circle. Variables that are not confounders or intermediate factors are represented in a grey circle. Arrows indicate the direction of causal paths. SES: socio-economic status

# Supplementary Table S3. Details on multiple imputation (n=9679).

| **Variables of interest** | **Type** | **Models for imputation** | **Missing values**  **n (%)** |
| --- | --- | --- | --- |
| Allergic and respiratory clusters | Categorical  *(5 categories)* | No missing data | 0 (0.0%) |
| Diet Quality score | Continuous | No missing data | 0 (0.0%) |
| PANDiet score | Continuous | No missing data | 0 (0.0%) |
| Fruits | Continuous | No missing data | 0 (0.0%) |
| Vegetables | Continuous | No missing data | 0 (0.0%) |
| Legumes | Categorical  *(3 categories)* | No missing data | 0 (0.0%) |
| Starch and grains | Continuous | No missing data | 0 (0.0%) |
| Nuts | Binary | No missing data | 0 (0.0%) |
| Milk and dairy products | Categorical  *(3 categories)* | No missing data | 0 (0.0%) |
| Fish and shellfish | Categorical  *(3 categories)* | No missing data | 0 (0.0%) |
| Red meat | Binary | No missing data | 0 (0.0%) |
| Processed meat | Binary | No missing data | 0 (0.0%) |
| Poultry | Continuous | No missing data | 0 (0.0%) |
| Sugar-sweetened beverages | Continuous | No missing data | 0 (0.0%) |
| Size of maternity unit | Ordinal  *(5 categories)* | No missing data | 0 (0.0%) |
| Period of enrolment | Categorical  *(4 categories)* | No missing data | 0 (0.0%) |
| Child’s sex | Binary | No missing data | 0 (0.0%) |
| Living area | Binary | No missing data | 0 (0.0%) |
| Region | Categorical  *(9 categories)* | No missing data | 0 (0.0%) |
| Total energy intake | Continuous | No missing data | 0 (0.0%) |
| Maternal age at delivery | Continuous | Linear regression | 1 (0.0%) |
| Maternal smoking status during pregnancy | Ordinal  *(4 categories)* | Logistic regression | 79 (0.8%) |
| Physical activity level during pregnancy | Continuous | Linear regression | 80 (0.8%) |
| Maternal pre-pregnancy BMI | Continuous | Linear regression | 97 (1.0%) |
| Number of older children | Ordinal  *(3 categories)* | Logistic regression | 110 (1.1%) |
| Maternal migration status | Categorical  *(3 categories)* | Multinomial regression | 141 (1.5%) |
| Family history of allergy | Binary | Logistic regression | 150 (1.6%) |
| Maternal education level | Categorical  *(5 categories)* | Multinomial regression | 275 (2.8%) |
| Household income | Continuous | Linear regression | 375 (3.3%) |

Multiple imputation on covariates was performed to address missing data. Missing values were considered as missing at random and five independent datasets were generated by Fully Conditional Specification method (SAS, MI procedure, FCS method). Missing values were imputed using linear regression for continuous variables, logistic regression for ordinal or binary variables and multinomial model for categorical variables. Pooled effects were estimates with MIANALYSE procedure (V9.4 SAS).

|  | **Allergic and respiratory multimorbidity clusters** | | | | |
| --- | --- | --- | --- | --- | --- |
|  | **Asymptomatic** | **Early wheeze without asthma** | **Asthma only** | **Allergies without asthma** | **Multi-allergic** |
|  | n=4178 | n=3281 | n=711 | n=659 | n=850 |
| Diet Quality score (per unit) | 13.0 ± 1.2 | 12.9 ± 1.2 | 12.9 ± 1.2 | 12.9 ± 1.2 | 13.0 ± 1.2 |
| PANDiet score (per units) | 56.0 ± 9.2 | 55.5 ± 9.0 | 55.7 ± 8.6 | 55.9 ± 9.3 | 56.2 ± 9.2 |
| Fruits (times/day) | 1.5 ± 1.7 | 1.4 ± 1.4 | 1.4 ± 1.4 | 1.4 ± 1.6 | 1.4 ± 1.5 |
| Vegetables (times/day) | 1.6 ± 1.3 | 1.6 ± 1.2 | 1.6 ± 1.3 | 1.6 ± 1.3 | 1.6 ± 1.2 |
| Legumes |  |  |  |  |  |
| <1 time/month | 80.7% (3372) | 83.6% (2742) | 82.1% (584) | 81.8% (539) | 82.5% (701) |
| 1-4 times/month | 14.8% (620) | 13.0% (425) | 13.2% (94) | 13.7% (90) | 13.9% (118) |
| >1 times/week | 4.5% (186) | 3.5% (114) | 4.6% (33) | 4.6% (30) | 3.6% (31) |
| Starch and grains (times/day) | 2.9 ± 1.2 | 2.8 ± 1.1 | 2.8 ± 1.2 | 2.9 ± 1.3 | 2.8 ± 1.2 |
| Nuts |  |  |  |  |  |
| No consumption | 67.6% (2823) | 68.3% (2241) | 69.3% (493) | 68.3% (450) | 67.6% (575) |
| Consumption | 32.4% (1355) | 31.7% (1040) | 30.7% (218) | 31.7% (209) | 32.4% (275) |
| Milk and dairy products |  |  |  |  |  |
| <3 times/day | 49.7% (2077) | 49.2% (1615) | 46.8% (333) | 54.0% (356) | 48.9% (416) |
| 3-4 times/day | 23.4% (978) | 24.0% (789) | 26.0% (185) | 21.4% (141) | 22.8% (194) |
| >4 times/day | 26.9% (1123) | 26.7% (877) | 27.1% (193) | 24.6% (162) | 28.2% (240) |
| Fish and shellfish |  |  |  |  |  |
| <1 time/week | 46.2% (1932) | 47.3% (1552) | 48.5% (345) | 47.8% (315) | 43.8% (372) |
| 1-2 times/week | 26.9% (1124) | 26.8% (878) | 27.8% (198) | 22.0% (145) | 29.1% (247) |
| >2 times/week | 26.9% (1122) | 25.9% (851) | 23.6% (168) | 30.2% (199) | 27.2% (231) |
| Red meat |  |  |  |  |  |
| <500 g/week | 73.9% (3086) | 72.5% (2378) | 72.7% (517) | 72.7% (479) | 75.4% (641) |
| ≥500 g/week | 26.1% (1092) | 27.5% (903) | 27.3% (194) | 27.3% (180) | 24.6% (209) |
| Processed meat |  |  |  |  |  |
| <150 g/week | 82.6% (3453) | 81.6% (2676) | 80.6% (573) | 80.3% (529) | 81.9% (696) |
| ≥150 g/week | 17.4% (725) | 18.4% (605) | 19.4% (138) | 19.7% (130) | 18.1% (154) |
| Poultry (g/week) | 151.0 ± 165.0 | 149.7 ± 141.9 | 141.2 ± 131.9 | 151.3 ± 137.0 | 160.3 ± 206.5 |
| Sweetened beverages (ml/day) | 460.5 ± 600.7 | 445.8 ± 554.8 | 454.6 ± 568.8 | 501.6 ± 635.4 | 480.3 ± 600.3 |
| Total energy intake (kcal/day) | 2166 ± 752 | 2163 ± 722 | 2154 ± 732 | 2173 ± 787 | 2179 ± 770 |

Supplementary Table S4. Maternal diet during pregnancy according to allergic and respiratory multimorbidity clusters in children (n=9679).

Values are % (n) or mean ± standard deviation.

# Supplementary Table S5. Allergic and respiratory characteristics by age of allergic and respiratory multimorbidity clusters (n=11,246).

|  | **Allergic and respiratory multimorbidity clusters** | | | | | |
| --- | --- | --- | --- | --- | --- | --- |
|  | **All** | **Asymptomatic** | **Early wheeze without asthma** | **Asthma only** | **Allergies without asthma** | **Multi-allergic** |
|  | n=11,246 | n=4841 | n=3785 | n=829 | n=804 | n=987 |
| Itchy rash reported at 2 months | 15.4% (1704) | 12.2% (574) | 15.3% (573) | 8.6% (70) | 24.6% (192) | 30.3% (295) |
| Itchy rash reported at 1 year | 25.7% (2805) | 20.4% (945) | 25.3% (941) | 17.9% (143) | 37.1% (286) | 50.4% (490) |
| Itchy rash reported at 2 years | 18.9% (2038) | 15.2% (702) | 17.6% (647) | 11.6% (93) | 27.7% (211) | 40.6% (385) |
| Itchy rash reported at 3.5 years | 16.8% (1775) | 13.1% (590) | 15.5% (556) | 11.5% (90) | 24.3% (179) | 38.3% (360) |
| Itchy rash reported at 5.5 years | 27.9% (3136) | 22.6% (1093) | 25.8% (976) | 19.5% (161) | 42.6% (341) | 57.3% (565) |
| Wheezing reported at 2 months | 5.7% (632) | 0.3% (15) | 10.5% (395) | 9.7% (79) | 6.3% (49) | 9.7% (94) |
| Wheezing reported at 1 year | 27.8% (3035) | 0.9% (43) | 50.7% (1887) | 57.0% (454) | 12.9% (99) | 56.8% (552) |
| Wheezing reported at 2 years | 19.6% (2111) | 0.9% (40) | 30.1% (1103) | 52.7% (423) | 9.8% (75) | 49.6% (470) |
| Wheezing reported at 3.5 years | 14.8% (1561) | 0.4% (19) | 18.5% (662) | 49.2% (386) | 7.3% (54) | 46.9% (440) |
| Wheezing reported at 5.5 years | 11.9% (1341) | 0.6% (27) | 11.4% (431) | 39.4% (327) | 8.2% (66) | 49.6% (490) |
| Night cough reported at 2 months | 24.7% (2727) | 18.0% (849) | 30.1% (1129) | 30.0% (244) | 24.5% (191) | 32.2% (314) |
| Night cough reported at 1 year | 68.9% (7513) | 52.4% (2431) | 84.4% (3140) | 78.8% (628) | 64.2% (494) | 84.4% (820) |
| Night cough reported at 2 years | 13.7% (1483) | 6.7% (310) | 17.0% (623) | 25.3% (203) | 11.8% (90) | 27.1% (257) |
| Night cough reported at 3.5 years | 20.2% (2131) | 11.6% (522) | 22.8% (816) | 36.4% (285) | 22.0% (162) | 36.8% (346) |
| Night cough reported at 5.5 years | 29.6% (3312) | 18.9% (912) | 29.9% (1127) | 43.6% (359) | 41.1% (329) | 59.6% (585) |
| Medication for asthma reported at 1 year | 23.8% (2597) | 0.9% (40) | 41.6% (1549) | 58.5% (466) | 4.9% (38) | 51.9% (504) |
| Medication for asthma reported at 2 years | 25.5% (2752) | 1.8% (85) | 39.7% (1457) | 69.4% (557) | 8.4% (64) | 62.1% (589) |
| Medication for asthma reported at 3.5 years | 21.6% (2276) | 1.3% (57) | 28.3% (1013) | 70.7% (554) | 8.3% (61) | 62.9% (591) |
| Consultation for asthma reported at 2 years | 2.1% (230) | 0.0% (0) | 0.0% (0) | 15.8% (127) | 0.0% (0) | 10.9% (103) |
| Consultation for asthma reported at 3.5 years | 2.2% (233) | 0.0% (0) | 0.0% (0) | 13.9% (109) | 0.1% (1) | 13.1% (123) |
| Consultation for asthma reported at 5.5 years | 15.5% (1739) | 0.7% (33) | 0.1% (2) | 97.3% (807) | 15.9% (127) | 78.3% (770) |
| Allergic conjunctivitis reported at 3.5 years | 23.1% (2439) | 17.6% (790) | 23.0% (823) | 17.2% (135) | 35.9% (264) | 45.5% (427) |
| Allergic conjunctivitis reported at 5.5 years | 24.1% (2714) | 18.3% (885) | 21.5% (815) | 16.6% (138) | 47.3% (380) | 50.3% (496) |
| Allergic rhinitis reported at 5.5 years | 12.1% (1366) | 2.7% (130) | 0.7% (25) | 11.8% (98) | 76.5% (615) | 50.5% (498) |
| Ever food allergies (0-5.5 years) | 7.4% (837) | 4.2% (205) | 5.2% (195) | 0.4% (3) | 18.7% (150) | 28.8% (284) |

Values are % (n).

# Supplementary Table S6. Adjusted associations of maternal diet quality during pregnancy with allergic and respiratory multimorbidity clusters in children from the complete-case sample (n=8939).

|  | **Allergic and respiratory multimorbidity clusters** | | | |
| --- | --- | --- | --- | --- |
|  | **Early wheeze without asthma** | **Asthma only** | **Allergies without asthma** | **Multi-allergic** |
| Diet Quality score (per unit) | 0.97 [0.93;1.02] | 1.00 [0.93;1.07] | 1.01 [0.93;1.09] | 1.03 [0.96;1.10] |
| PANDiet score (per 10 units) | 0.95 [0.90;1.01] | 1.00 [0.91;1.11] | 0.96 [0.86;1.07] | 1.06 [0.96;1.16] |
| Fruits (times/day) | 0.97 [0.93;1.00] | 1.02 [0.96;1.09] | 1.00 [0.94;1.07] | 0.96 [0.91;1.02] |
| Vegetables (times/day) | 1.02 [0.97;1.06] | 1.02 [0.94;1.10] | 1.03 [0.95;1.11] | 0.99 [0.92;1.07] |
| Legumes |  |  |  |  |
| <1 time/month | 1 [Ref] | 1 [Ref] | 1 [Ref] | 1 [Ref] |
| 1-4 times/month | **0.84 [0.72;0.97]** | 0.92 [0.72;1.17] | 0.95 [0.73;1.23] | 0.92 [0.73;1.15] |
| >1 times/week | 0.79 [0.61;1.02] | 0.93 [0.60;1.45] | 0.97 [0.62;1.51] | 0.82 [0.54;1.25] |
| Starch and grains (times/day) | 1.00 [0.95;1.05] | 0.97 [0.89;1.06] | 1.02 [0.93;1.11] | 0.96 [0.89;1.04] |
| Nuts (consumption vs no consumption) | 0.96 [0.87;1.07] | 1.01 [0.84;1.21] | 0.96 [0.80;1.16] | 0.86 [0.73;1.02] |
| Milk and dairy products |  |  |  |  |
| <3 times/day | 0.99 [0.88;1.12] | 0.82 [0.66;1.01] | 1.13 [0.90;1.42] | 0.99 [0.81;1.21] |
| 3-4 times/day | 1 [Ref] | 1 [Ref] | 1 [Ref] | 1 [Ref] |
| >4 times/day | 0.96 [0.84;1.11] | 0.93 [0.73;1.18] | 0.99 [0.76;1.28] | 1.06 [0.85;1.33] |
| Fish and shellfish |  |  |  |  |
| <1 time/week | 1.04 [0.92;1.17] | 1.07 [0.87;1.31] | 1.19 [0.95;1.50] | 0.84 [0.70;1.02] |
| 1-2 times/week | 1 [Ref] | 1 [Ref] | 1 [Ref] | 1 [Ref] |
| >2 times/week | 0.99 [0.87;1.14] | 0.91 [0.72;1.15] | **1.40 [1.09;1.79]** | 0.89 [0.72;1.10] |
| Red meat (≥500 vs <500 g/week) | 1.05 [0.94;1.18] | 1.08 [0.88;1.31] | 1.03 [0.83;1.27] | 0.89 [0.74;1.08] |
| Processed meat (≥150 vs <150 g/week) | 1.04 [0.92;1.19] | 1.06 [0.84;1.33] | 1.24 [0.98;1.56] | 1.03 [0.83;1.27] |
| Poultry (per 100 g/week) | 1.00 [0.96;1.03] | 0.97 [0.91;1.03] | 1.01 [0.95;1.06] | 1.03 [0.98;1.08] |
| Sweetened beverages (per 200 ml/day) | 0.99 [0.97;1.01] | 0.99 [0.96;1.03] | 1.01 [0.97;1.04] | 1.01 [0.98;1.04] |

Cluster of reference: “asymptomatic”. Results with p-value < 0.05 are in bold text. Adjusted OR [95%CI] from multinomial logistic regressions. OR, odds ratio; CI, confidence interval. Each dietary exposure was considered in a separate model. Models for diet quality scores are adjusted for maternal characteristics (age at delivery, education level, household income, migration status, smoking status during pregnancy, pre-pregnancy body mass index (BMI), physical activity during pregnancy, parity, living area (urban/rural), region and total energy intake), child characteristics (sex and family history of atopy), and study design characteristic (period of enrolment, size of maternity unit). For food groups, models were also adjusted on the PANDiet score.

# Supplementary Table S7. Weighted associations of maternal diet quality during pregnancy with allergic and respiratory multimorbidity clusters in children from the main sample (n=9677).

|  | **Allergic and respiratory multimorbidity clusters** | | | |
| --- | --- | --- | --- | --- |
|  | **Early wheeze without asthma** | **Asthma only** | **Allergies without asthma** | **Multi-allergic** |
| Diet Quality score (per unit) | 0.97 [0.92;1.03] | 0.98 [0.89;1.07] | 1.00 [0.92;1.10] | 0.98 [0.90;1.07] |
| PANDiet score (per 10 units) | 0.94 [0.87;1.00] | 1.03 [0.91;1.16] | 1.02 [0.89;1.18] | 1.01 [0.90;1.13] |
| Fruits (times/day) | 0.98 [0.94;1.03] | 1.01 [0.93;1.09] | 0.98 [0.92;1.05] | 0.97 [0.90;1.04] |
| Vegetables (times/day) | 1.00 [0.94;1.05] | 1.01 [0.91;1.12] | **1.09 [1.00;1.18]** | 0.97 [0.89;1.06] |
| Legumes |  |  |  |  |
| <1 time/month | 1[Ref] | 1[Ref] | 1[Ref] | 1[Ref] |
| 1-4 times/month | 0.87 [0.73;1.05] | 0.81 [0.60;1.11] | 0.78 [0.57;1.07] | 0.81 [0.61;1.08] |
| >1 times/week | 0.85 [0.62;1.18] | 1.37 [0.83;2.28] | 1.25 [0.74;2.12] | 0.68 [0.39;1.19] |
| Starch and grains (times/day) | 0.97 [0.91;1.03] | 0.93 [0.84;1.04] | 0.99 [0.89;1.10] | **0.90 [0.82;0.99]** |
| Nuts (consumption vs no consumption) | 1.00 [0.88;1.14] | 1.15 [0.91;1.44] | 1.07 [0.85;1.34] | 0.91 [0.74;1.12] |
| Milk and dairy products |  |  |  |  |
| <3 times/day | 0.93 [0.79;1.09] | 0.95 [0.73;1.25] | 1.01 [0.76;1.34] | 0.97 [0.75;1.25] |
| 3-4 times/day | 1[Ref] | 1[Ref] | 1[Ref] | 1[Ref] |
| >4 times/day | 1.00 [0.84;1.20] | 1.03 [0.76;1.39] | 0.89 [0.64;1.22] | 1.00 [0.75;1.33] |
| Fish and shellfish |  |  |  |  |
| <1 time/week | 1.06 [0.91;1.23] | 1.05 [0.81;1.36] | 1.20 [0.92;1.58] | 0.99 [0.78;1.25] |
| 1-2 times/week | 1[Ref] | 1[Ref] | 1[Ref] | 1[Ref] |
| >2 times/week | 1.06 [0.90;1.26] | 0.88 [0.65;1.19] | 1.33 [0.98;1.80] | 1.00 [0.77;1.30] |
| Red meat (≥500 vs <500 g/week) | 1.04 [0.90;1.20] | 0.98 [0.75;1.26] | 1.10 [0.86;1.41] | 0.81 [0.64;1.03] |
| Processed meat (≥150 vs <150 g/week) | 1.09 [0.92;1.28] | 1.18 [0.88;1.57] | 1.26 [0.95;1.68] | 0.99 [0.77;1.27] |
| Poultry (per 100 g/week) | 0.99 [0.95;1.03] | 0.97 [0.90;1.04] | 0.98 [0.92;1.05] | 1.00 [0.95;1.06] |
| Sweetened beverages (per 200 ml/day) | 0.99 [0.97;1.02] | 1.00 [0.96;1.04] | 1.02 [0.98;1.06] | 1.02 [0.99;1.06] |

Cluster of reference: “asymptomatic”. Results with p-value < 0.05 are in bold text. Adjusted OR [95%CI] from multinomial logistic regressions. OR, odds ratio; CI, confidence interval. Each dietary exposure was considered in a separate model. Models for diet quality scores are adjusted for maternal characteristics (age at delivery, education level, household income, maternal migration history, smoking status during pregnancy, pre-pregnancy body mass index (BMI), physical activity during pregnancy, number of older children, living area (urban/rural), region and total energy intake), child characteristics (sex and family history of atopy), and study design characteristic (period of enrolment, size of maternity unit). For food groups, models were also adjusted on the PANDiet score.

|  | **Allergic and respiratory multimorbidity clusters** | | | | | | | |
| --- | --- | --- | --- | --- | --- | --- | --- | --- |
|  | **Early wheeze without asthma** | | **Asthma only** | | **Allergies without asthma** | | **Multi-allergic** | |
|  | aOR(95%CI) | q-value | aOR(95%CI) | q-value | aOR(95%CI) | q-value | aOR(95%CI) | q-value |
| Fruits (times/day) | 0.97 [0.94;1.00] | 0.50 | 1.00 [0.95;1.06] | 0.93 | 1.00 [0.94;1.06] | 0.95 | 0.96 [0.90;1.01] | 0.51 |
| Vegetables (times/day) | 1.01 [0.97;1.05] | 0.84 | 1.01 [0.93;1.08] | 0.93 | 1.04 [0.96;1.11] | 0.90 | 0.98 [0.92;1.05] | 0.71 |
| Legumes |  |  |  |  |  |  |  |  |
| <1 time/month | 1 [Ref] |  | 1 [Ref] |  | 1 [Ref] |  | 1 [Ref] |  |
| 1-4 times/month | 0.86 [0.75;0.98] | 0.37 | 0.89 [0.70;1.14] | 0.93 | 0.92 [0.72;1.18] | 0.90 | 0.91 [0.73;1.13] | 0.68 |
| >1 times/week | 0.82 [0.64;1.04] | 0.50 | 1.11 [0.75;1.65] | 0.93 | 0.99 [0.66;1.49] | 0.95 | 0.80 [0.54;1.20] | 0.57 |
| Starch and grains (times/day) | 0.99 [0.95;1.04] | 0.84 | 0.97 [0.89;1.05] | 0.93 | 1.01 [0.93;1.09] | 0.90 | 0.96 [0.89;1.03] | 0.51 |
| Nuts (consumption vs no consumption) | 0.96 [0.86;1.06] | 0.84 | 0.99 [0.83;1.18] | 0.93 | 0.94 [0.79;1.12] | 0.95 | 0.86 [0.73;1.01] | 0.57 |
| Milk and dairy products |  |  |  |  |  |  |  |  |
| <3 times/day | 0.97 [0.86;1.10] | 0.84 | 0.84 [0.68;1.03] | 0.80 | 1.16 [0.93;1.44] | 0.66 | 0.99 [0.81;1.20] | 0.93 |
| 3-4 times/day | 1 [Ref] |  | 1 [Ref] |  | 1 [Ref] |  | 1 [Ref] |  |
| >4 times/day | 0.98 [0.85;1.12] | 0.84 | 0.93 [0.74;1.18] | 0.93 | 0.98 [0.76;1.25] | 0.95 | 1.06 [0.85;1.32] | 0.71 |
| Fish and shellfish |  |  |  |  |  |  |  |  |
| <1 time/week | 1.04 [0.93;1.16] | 0.84 | 1.03 [0.84;1.25] | 0.93 | 1.17 [0.95;1.46] | 0.66 | 0.86 [0.71;1.03] | 0.51 |
| 1-2 times/week | 1 [Ref] |  | 1 [Ref] |  | 1 [Ref] |  | 1 [Ref] |  |
| >2 times/week | 1.00 [0.88;1.14] | 0.95 | 0.85 [0.68;1.07] | 0.80 | 1.37 [1.08;1.74] | 0.12 | 0.92 [0.75;1.13] | 0.68 |
| Red meat (≥500 vs <500 g/week) | 1.06 [0.95;1.18] | 0.84 | 1.04 [0.86;1.26] | 0.93 | 1.04 [0.85;1.27] | 0.95 | 0.87 [0.73;1.05] | 0.51 |
| Processed meat (≥150 vs <150 g/week) | 1.05 [0.92;1.19] | 0.84 | 1.09 [0.88;1.36] | 0.93 | 1.19 [0.96;1.49] | 0.66 | 1.01 [0.82;1.24] | 0.93 |
| Poultry (per 100 g/week) | 1.00 [0.97;1.03] | 0.84 | 0.96 [0.90;1.02] | 0.80 | 1.00 [0.94;1.05] | 0.95 | 1.02 [0.98;1.07] | 0.57 |
| Sweetened beverages (per 200 ml/day) | 0.99 [0.97;1.01] | 0.84 | 1.00 [0.96;1.03] | 0.93 | 1.01 [0.98;1.04] | 0.90 | 1.01 [0.98;1.04] | 0.71 |

# Supplementary Table S8. Adjusted associations of maternal food groups consumption with allergic and respiratory multimorbidity clusters in children (n=9679), after correction for multiple testing.

Cluster of reference: “asymptomatic”. Adjusted OR [95%CI] from multinomial logistic regressions. OR, odds ratio; CI, confidence interval. Each dietary exposure was considered in a separate model. Models are adjusted for maternal characteristics (age at delivery, education level, household income, migration status, smoking status during pregnancy, pre-pregnancy body mass index (BMI), physical activity during pregnancy, parity, living area (urban/rural), region, total energy intake, and PANDiet score), child characteristics (sex, family history of allergy, breastfeeding duration, late introduction of allergenic food), and study design characteristic (period of enrolment, size of maternity unit). Results were corrected for multiple testing using the False Discovery Rate procedure, a q-value < 0.1 was considered significant.
